# Supplementary figures and images for: Safety profile of inactivated COVID-19 in healthy adults aged ≥ 18 years: A passive surveillance in Indonesia
Source: PLoS One. 2023 Oct 12;18(10):e0286484. doi: 10.1371/journal.pone.0286484 (PMC10569643; doi:10.1371/journal.pone.0286484)

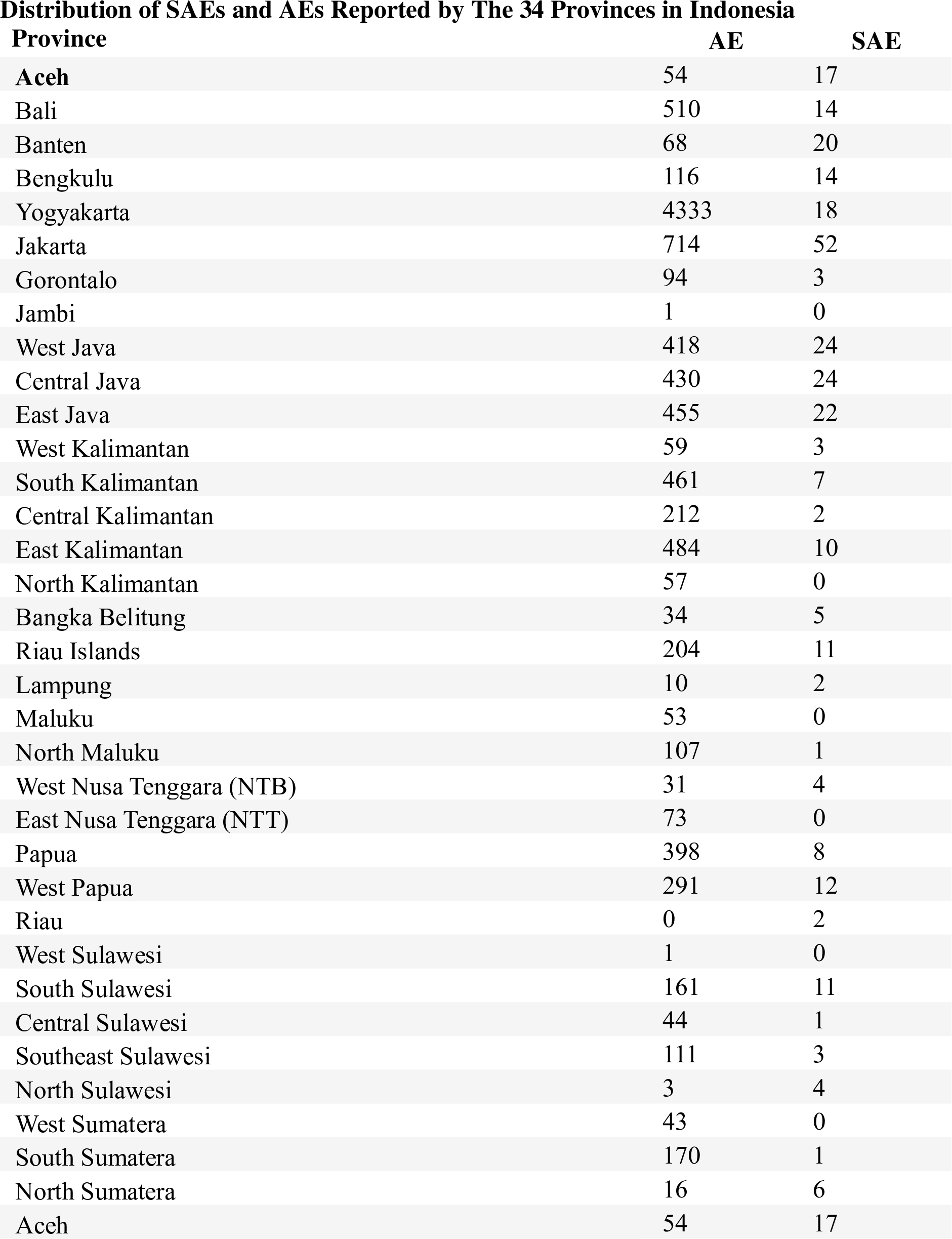

Supplement: S1 File — (ZIP) [file pone.0286484.s001.zip › PACE Corrected/Supplementary Files 1 (Data Table) (1).tif]

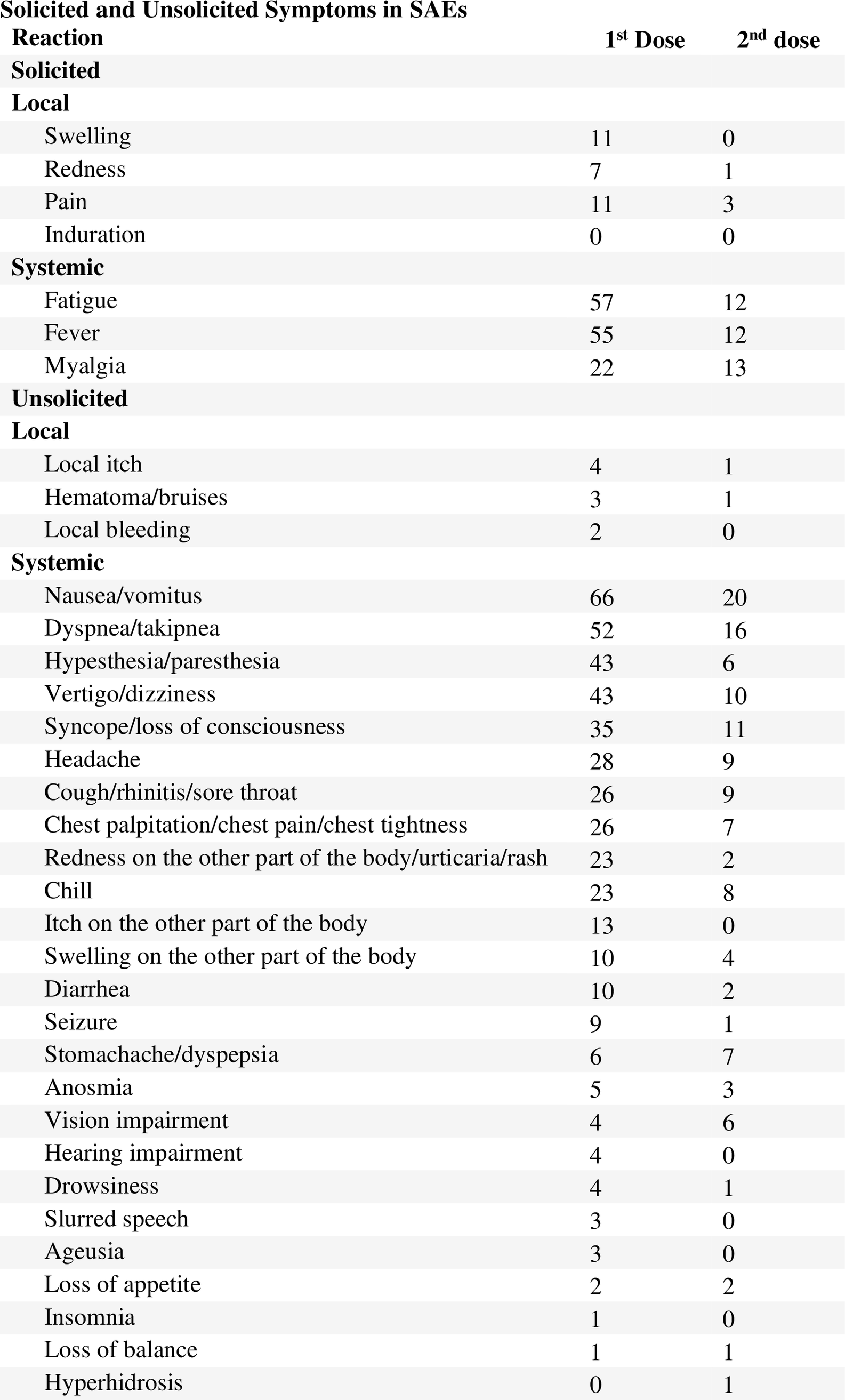

Supplement: S1 File — (ZIP) [file pone.0286484.s001.zip › PACE Corrected/Supplementary Files 1 (Data Table) (1).tif]

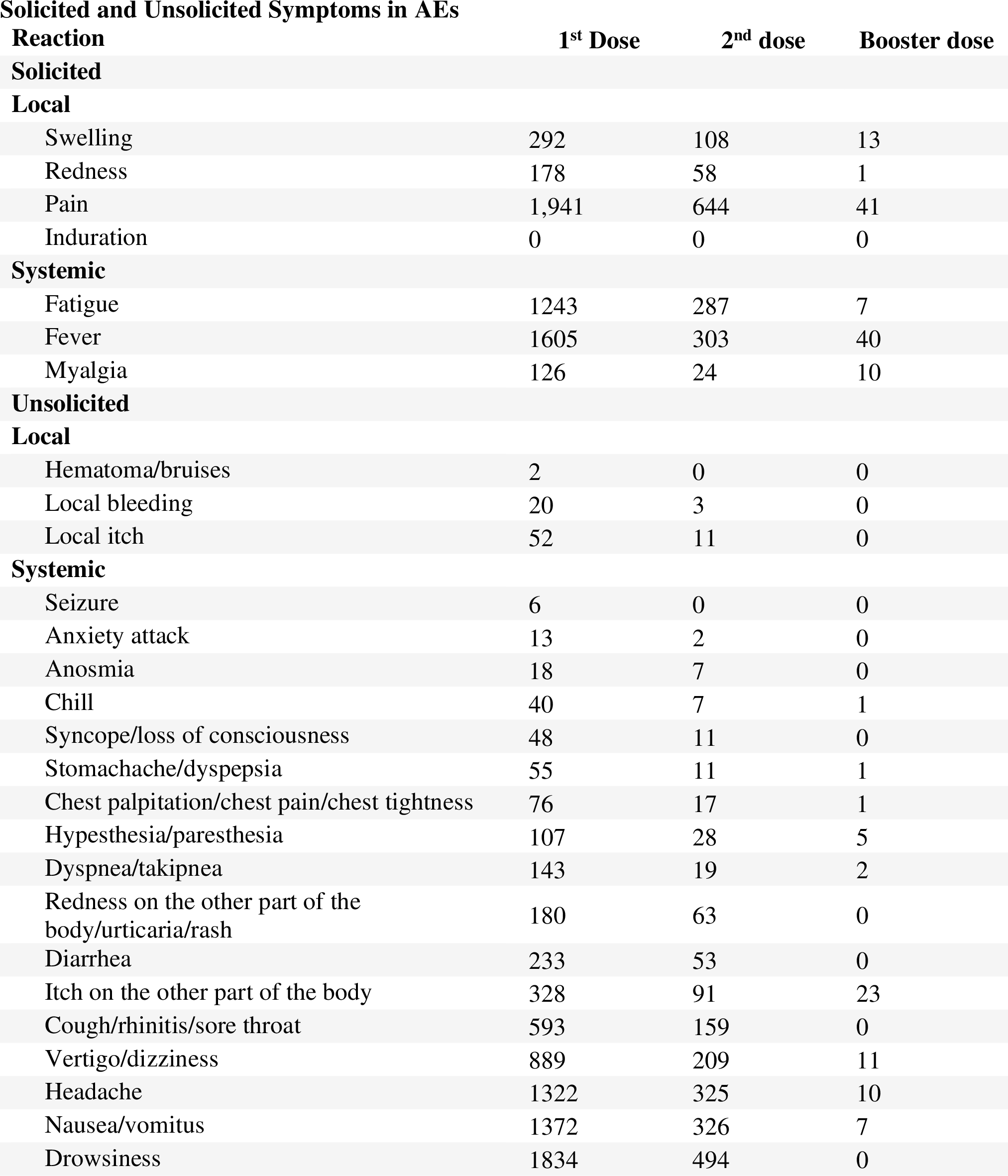

Supplement: S1 File — (ZIP) [file pone.0286484.s001.zip › PACE Corrected/Supplementary Files 1 (Data Table) (1).tif]

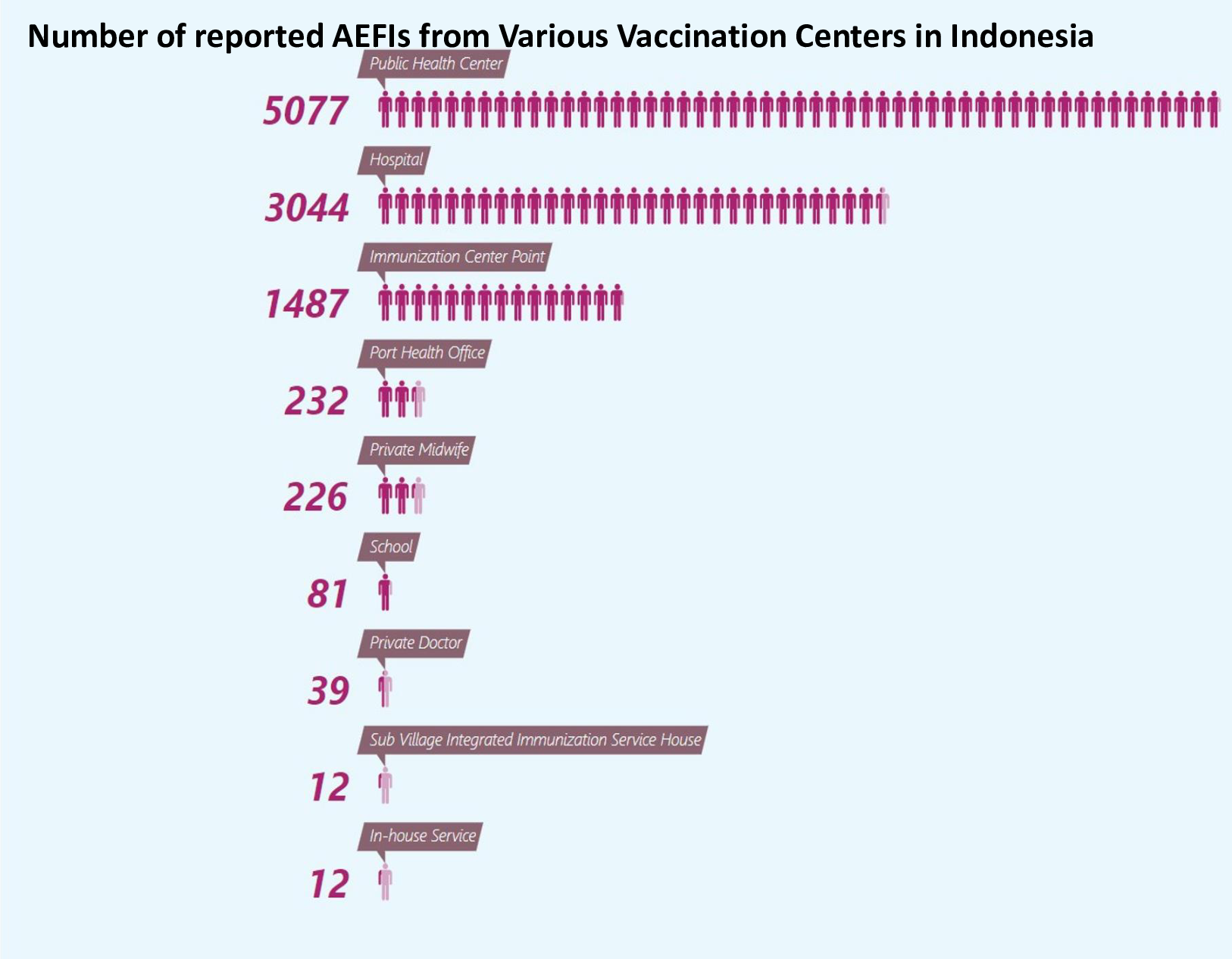

Supplement: S1 File — (ZIP) [file pone.0286484.s001.zip › PACE Corrected/Supplementary Files 2 (1).tif]
