# Supplementary material for: Safety profile of inactivated COVID-19 in healthy adults aged ≥ 18 years: A passive surveillance in Indonesia
Source: PLoS One. 2023 Oct 12;18(10):e0286484. doi: 10.1371/journal.pone.0286484 (PMC10569643; doi:10.1371/journal.pone.0286484)
Supplement: Attachment — Submitted filename: Response Letter to Editor and Reviewer.docx [file pone.0286484.s002.docx]

Mohammad-Reza Malekpour, M.D.

Academic Editor

Plos One

April 18^th^ 2023

Dear Academic Editor, Dr. Malekpour:

Subject: Submission of Revised Paper Safety Profile of An Inactivated SARS-CoV-2 Vaccine (Sinovac/CoronaVac) in Healthy Adults aged ≥ 18 Years: a Passive Surveillance in Indonesia PLOS ONE [PONE-D-23-02102] - [EMID:fb8ac8d3d8d39754]

We are grateful for your kind and generous feedback in the email dated March 8^th^, 2022 enclosing the reviewer's comments. Your comments provided valuable insights to refine its contents and analysis. We have carefully reviewed the comments and have revised our manuscript accordingly. Our responses are listed below. Changes to the manuscripts are shown in the highlight or with track changes in the document.

Thank you for giving us the opportunity to submit a revised draft of the manuscript "Safety Profile of An Inactivated SARS-CoV-2 Vaccine (Sinovac/CoronaVac) in Healthy Adults aged ≥ 18 Years: a Passive Surveillance in Indonesia" for publication in the PlosOne. We sincerely hope the revised document will answer all the comments and be suitable for publication. We are looking forward to hearing from you in due course.

Sincerely yours,

Nastiti Kaswandani, M.D., Paediatrician, PhD

Bernie Endyarni Medise, M.D., Paediatrician, PhD

Elcha Leonard, M.D.

And researcher’s team

**Responses to Reviewer 1**

- Introduction, lines 21-24: the statistics need to be updated.

As suggested by the reviewer, we have updated the latest statistical data on COVID-19 cases and deaths, and the changes can be found in lines 21-25

- Materials and methods: the first three subheadings can be merged.

We have merged the first three subheadings in the material and methods section (lines 62-74)

- In the study results, it is reported that the female gender accounted for more adverse events after first, second, or booster dose. But is there any proof that females weren't more vaccinated than males? For concluding that adverse events are more common among females, we are going to need such fractions: females having symptoms/total vaccinated females Vs. Males having symptoms/total vaccinated males.

Thank you for this suggestion. It would have been interesting to explore this aspect. Unfortunately, this would not be possible in our study because there is no specific national data for total Sinovac/Coronavac doses provided by gender group.

**Responses to Reviewer 2**

- The abstract first line ‘…………is one of the methods to prevent…..’ may be reframed

We have reframed the first line in the abstract (lines 2-3)

- Sentence in lines 52-55 is not clear.

We agree with the reviewer's assessment. We have revised the suggested part in the manuscript to make it more knowledgeable to the readers. (lines 56-57)

- Line 55' world' not 'word'

Thank you for pointing this out. The reviewer is correct, and we have changed it to "world"

- Authors may consider adding the 'Study design' section briefly.

Thank you for your suggestion. We have added the study design section and tried to merge it into the material and methods section. (lines 62-78)

- The sum of 'Total Serious AEFI reported (n cases)' in Table 1 is not correct.

We agree with the reviewer's assessment. Accordingly, we have revised the total Serious AEFIs reported (n cases) throughout the manuscript.

- Fix lines 143 and 144: 5.104 instead of 5,104 and 1.391 instead of 1,391.

While we appreciate the reviewer's feedback, we respectfully disagree. The 5,104 and 1,391 stated in the manuscript mean to show the total number of cases of AE reported in female and males group, respectively.

- Line 176: remove , with . in data

Thank you for pointing this out. We have changed it according to the reviewer's suggestion.

**Responses to Reviewer 3**

- Given that a major strength of the study is the use of the national surveillance data, the analysis should go beyond provides descriptive outputs about AEFI but include an assessment of the quality or completeness of the data. Authors mention that all health facilities are required by policy to report into the system, but there is no analysis to show if indeed all facilities providing the COVID-19 vaccine are reporting. This is important to inform the interpretation of the AEFI incidence rate presented in the analysis for the whole country.

We think this is an excellent suggestion. We have portrayed the SAE/AE reports from all of the provinces in Indonesia in Figure 1. We have also added the total number of reported AEFIs from various vaccination centers in Indonesia, listed in Supplementary Files 2.

- It is important to report the proportion of COVID-19 vaccines in use that was Sinovac. If other vaccines were in use at the same time, please report them and their percentages.

We agreed with the reviewer's suggestion. We have added the total doses of COVID-19 vaccination given in Indonesia simultaneously with various platforms and their percentages (lines 33-36).

- The methods section does not describe their approach to analysis. Authors should revise this section and include what analysis was done and what statistically tests were done - if any. There should also be a statement around ethical review - if one was sought or done, if not why not.

As suggested by the reviewer, we have incorporated the analysis and statistical test that have been done. We have also added the ethical review of this study (lines 75-78)

- The discussion section should do a bit more than report what was found, it should interpret the results and tell the reader the implications of the findings. For example, authors could discuss the AEFI rates in relation to expected rates or discuss any potential challenge with the data that could affect the inference drawn from their findings.

Thank you for your exquisite opinion. We have tried refining the discussion section and further explaining potential challenges in this study.

- Grammatical errors and fix punctuation

Thank you for pointing this out. As the reviewers suggested, we have fixed this study's grammatical errors and punctuation.
